# The Association Between Cancer Incidence and Heart Failure: A Systematic Review and Meta-Analysis

**DOI:** 10.3390/diagnostics16132016

**Published:** 2026-06-28

**Authors:** Sabah Mohammed Al-Harazi, Barani Karikalan, Meram Azzani, Mohammed Shahjahan Kabir, Deepa Anbazhagan, Prarthana Kalerammana Gopalakrishna, Rajesh Thangarajan, Amal Shediwah, Nahlah Abduljaleel Alsaidi, Rola Ali-Saeed, Lubna Shirin, Mohamed Afiq Hidayat Zailani

**Affiliations:** 1 Early Clinical Exposure and Professional Personal Development, Faculty of Medicine, MAHSA University, Jenjarom 42610, Selangor, Malaysia; mohammedsabah@mahsa.edu.my; 2 Department of Pathology, Faculty of Medicine, MAHSA University, Jenjarom 42610, Selangor, Malaysia; barani@mahsa.edu.my; 3 Department of Public Health Medicine, Faculty of Medicine, Universiti Teknologi MARA (UiTM), Sungai Buloh 47000, Selangor, Malaysia; meram@uitm.edu.my; 4 Department of Medicine, International Medical School, Management and Science University (MSU), Shah Alam 40100, Selangor, Malaysia; drkabir557@gmail.com; 5 Department of Microbiology, International Medical School, Management and Science University (MSU), Shah Alam 40100, Selangor, Malaysia; deepa_anbazhagan@msu.edu.my; 6 Physiology Discipline, Human Biology Department, School of Medicine, IMU University, Kuala Lumpur 57000, Malaysia; prarthana@imu.edu.my; 7 Department of Anatomy, International Medical School, Management and Science University (MSU), Shah Alam 40100, Selangor, Malaysia; rajesh_thangarajan@msu.edu.my; 8 Department of Pathology, College of Medicine, Najran University, Najran 61441, Saudi Arabia; amshediwah@nu.edu.sa; 9 Department of Community Medicine, Faculty of Medicine, MAHSA University, Jenjarom 42610, Selangor, Malaysia; nahlah.a@mahsa.edu.my; 10 Department of Preclinical Sciences, Faculty of Dentistry, MAHSA University, Jenjarom 42610, Selangor, Malaysia; rolaali@mahsa.edu.my; 11 Department of Anatomy, Faculty of Medicine, Universiti Teknologi MARA (UiTM), Sungai Buloh 47000, Selangor, Malaysia; lubnashirin@uitm.edu.my; 12 Department of Pathology, Faculty of Medicine, Universiti Kebangsaan Malaysia (UKM), Kuala Lumpur 56000, Malaysia

**Keywords:** heart failure, cancer incidence, systematic review, meta-analysis, hazard ratio, cardio-oncology, molecular genetics, pathology, Genomics

## Abstract

**Background/Objectives**: Heart failure (HF) and cancers share common molecular and pathophysiological mechanisms. However, the association between HF and the risk of developing cancer remains unclear. This systematic review and meta-analysis evaluates the association between HF and the risk of cancer incidence by pooling data from studies that compared cancer incidence in HF patients with non-HF controls. Additionally, it quantifies pooled hazard ratios (HRs) for site-specific cancers. **Methods**: A systematic literature search was conducted across four databases until March 2025 (PROSPERO: CRD420251001541). Seven studies involving over 1.6 million participants were included. Three studies provided direct HF versus non-HF comparisons. Pooled HRs and their corresponding 95% confidence intervals (CIs) were calculated utilising random-effects models. **Results**: The pooled estimate for overall cancer in HF was not significant (HR 1.32; 95% CI: 0.94–1.85; *p* = 0.11). However, site-specific analyses revealed significantly elevated risks for lung cancer (HR 1.66; 95% CI: 1.27–2.16), urinary tract cancers (HR 1.66; 95% CI: 1.51–1.82), haematological cancers (HR 1.47; 95% CI: 1.12–1.94), and colorectal cancer (HR 1.38; 95% CI: 1.04–1.83). The associations with breast and prostate cancers were not significant. Notably, substantial heterogeneity was observed across most cancer sites (I^2^ = 96–98%), apart from urinary tract cancers (I^2^ = 0%). **Conclusions**: Although the pooled estimate for overall cancer was not significant, the significant findings for specific cancer sites suggest the need for enhanced cancer surveillance in patients with HF. Future research should focus on standardising definitions of HF, ensuring consistent adjustment for confounders, and stratifying data by HF phenotype.

## 1. Introduction

Cardiovascular diseases (CVDs) encompass a diverse range of conditions that impact the cardiovascular system and remain the leading cause of death worldwide. Among these, heart failure (HF) is frequently the ultimate result of various cardiovascular disorders and significantly contributes to negative clinical outcomes. Research suggests that individuals with CVD may face a heightened risk of developing cancer, which may stem from two main hypotheses: the influence of inflammation, oxidative stress, and molecular and genetic predisposition in promoting both conditions, or the possibility that HF acts as an oncogenic state via the neurohormonal activation of the renin–angiotensin–aldosterone system (RAAS) and the release of serine protease inhibitor (SERPINA3), which aids in tumour growth [1].

Furthermore, inflammatory cytokines and metabolic abnormalities related to glucose oxidation, aerobic glycolysis, and de novo lipogenesis have been linked to the association between HF and cancer [2,3]. The association between HF and cancer has emerged as a vital area of research, with growing evidence indicating that individuals with HF might be at an increased risk of developing various types of cancer [4,5,6]. A significant observational study involving over 200,000 individuals indicated that patients with HF displayed a considerably higher incidence of cancer compared to those without HF [4]. The cancer incidence was 25.7% in HF patients versus 16.2% in controls over a 10-year period, resulting in a hazard ratio (HR) of 1.76 for the development of cancer in HF patients [4].

The HR for women and men was found to be 1.85 and 1.69, respectively. This trend was consistent across various types of cancer. The highest risk was observed for cancers of the lip, oral cavity, and pharynx, which showed an HR of 2.10, followed by respiratory system cancer, which had an HR of 1.91. The HRs for different cancer sites are as follows: 1.86 for female genital organ cancer, 1.83 for skin tumours, 1.77 for lymphoid and haematopoietic tissue cancer, 1.75 for digestive tract cancer, 1.67 for breast cancer, 1.64 for genitourinary tract cancer, and 1.52 for male genital organ cancer [4].

In a case–control study investigating the risk of cancer incidence among patients with HF, which comprised 961 HF patients and 961 controls, it was found that those with HF had a 60% increased risk of developing cancer, as indicated by a hazard ratio (HR) of 1.60, compared to the non-HF controls. The findings were corroborated after adjusting for several prevalent risk factors associated with both cancer and HF, including body mass index, smoking habits, and comorbidities [7]. Among the 244 identified cases of cancer, there were 48 related to the digestive system, 46 concerning male reproductive organs, 39 classified as haematologic cancers, 24 involving breast cancers, 20 associated with respiratory cancers, 19 linked to urinary cancers, 7 pertaining to female reproductive cancers, 7 identified as skin cancers, and 34 categorised as other types of cancers [7].

A prospective cohort study involving 596 patients with HF and 596 controls was conducted to further investigate the risk of cancer among a homogeneous group of myocardial infarction (MI) survivors. The findings of this study indicated an increased risk of cancer in patients who experienced HF following MI [7]. These results are consistent with evidence from another study, which reported that patients with HF exhibited a notable increase in the incidence of cancer, with an HR of 1.43 compared to individuals without HF [6]. The increase was especially significant for certain cancers, including lung cancer (HR = 1.89), breast cancer (HR = 1.28), haematological cancers (HR = 1.63), and colorectal cancer (HR = 1.32) [6]. The incidence of prostate cancer was similar in both groups (HR = 0.97) [6].

It has been found that HF is related to a greater incidence of cancer, even when secondary analyses are controlled for biases caused by heterogeneity across studies [5]. After the age of 45, the lifetime risk of HF ranges from 20% to 45%. There is an increasing incidence of heart failure with preserved ejection fraction (HFpEF), while the incidence of HF with reduced ejection fraction (HFrEF) is decreasing [8]. This expanding burden of HF has been accompanied by growing evidence that patients with HF have a significantly higher risk of developing various site-specific cancers than those without HF. The elevated risks span multiple body systems, with oral cancers presenting an HR of 2.10 [4]. The respiratory system and lungs exhibited similarly elevated risks (HR = 1.89–1.91), along with a notable increase in haematological malignancies (HR = 1.63–1.77) [4,6]. Additionally, malignancies of the gastrointestinal tract showed elevated risks (HR = 1.75), including colorectal cancer (HR = 1.32) [4,6].

Among breast cancer cases in HF patients, there was a moderate but consistent increase (HR = 1.28–1.67) [4,6]. Overall, genitourinary cancers are more prevalent in this population (HR = 1.64), particularly cancers of the female genital organs, which have a higher HR of 1.86 [4]. In contrast, prostate cancer does not demonstrate a strong association with HF, showing an HR of 0.97 [6]. Additionally, the incidence of skin tumours is more pronounced in HF populations (HR = 1.83) [4]. These findings suggest a systemic connection between HF and cancer development, likely attributed to shared pathophysiological mechanisms, including chronic inflammation, immune dysregulation, metabolic remodelling, and neurohormonal activation. Collectively, this growing body of evidence points out the necessity of integrated cardio-oncology surveillance for managing patients with HF [9,10].

Radiation-induced heart failure (RIHF) is a well-known late-stage consequence of thoracic radiation, especially among survivors of breast cancer, lymphoma, lung cancer, and other mediastinal malignancies. Endothelial injury, microvascular dysfunction, chronic inflammation, oxidative stress, and myocardial fibrosis all contribute to accelerated atherosclerosis, ventricular remodelling, diastolic dysfunction, valvular disease, and subsequently, heart failure. Clinical signs may appear years to decades after radiotherapy, with the risk increasing with cardiac radiation dose and concurrent exposure to cardiotoxic medications such as anthracyclines or HER2-targeted drugs. Cancer survivors who have been exposed to thoracic irradiation should undergo long-term cardiovascular surveillance, which includes clinical examination, echocardiography, and risk factor evaluation. Management should follow current HF guidelines, which include guideline-directed medical therapy and intensive control of modifiable cardiovascular risk factors. Patients with significant coronary, valvular, or advanced myocardial disease may require specialized cardio-oncology care, including revascularization, valve interventions, device therapy, or other advanced HF treatment. Nonetheless, preventive efforts remain crucial, with an emphasis on minimizing cardiac radiation exposure using contemporary radiotherapy techniques and adhering to cardiac dose limitations.

Anticoagulation in patients with concurrent HF and active cancer presents unique clinical challenges due to the interplay between hypercoagulability, comorbidities, and treatment-related factors. Vitamin K antagonists (VKAs), such as warfarin, are often suboptimal because achieving stable international normalized ratio (INR) control is difficult. This instability arises from multiple factors common in cancer patients, including drug–drug interactions with chemotherapy, fluctuating nutritional intake (especially vitamin K), impaired hepatic metabolism, and frequent hospitalizations.

Low-molecular-weight heparins have traditionally been preferred in cancer-associated thrombosis due to their predictable dose–response relationship, minimal monitoring requirements, and lower interaction potential. More recently, direct oral anticoagulants (DOACs), including apixaban, rivaroxaban, and edoxaban, have emerged as effective alternatives. DOACs offer fixed dosing, rapid onset of action, and fewer dietary restrictions, improving patient convenience and adherence. However, anticoagulants (i.e., VKAs, LMWH, DOACs) are used to manage thrombosis, which is common in both heart failure and cancer, and current evidence does not support a causal role in promoting cancer development.

This systematic review and meta-analysis aimed to evaluate the association between HF and the risk of cancer incidence by pooling data from cohort studies that directly compare cancer incidence between HF patients and non-HF controls. Additionally, the study aimed to quantify pooled HRs for specific types of cancers, including lung, colorectal, breast, prostate, haematological, and urinary tract cancers, as well as to provide a qualitative narrative synthesis of studies that were excluded from the meta-analysis due to incompatible outcome metrics or the absence of a direct non-HF comparator.

## 2. Materials and Methods

### 2.1. Study Design

This systematic review and meta-analysis was performed following the Preferred Reporting Items for Systematic Reviews and Meta-Analyses (PRISMA) guidelines [11]. The study protocol was registered in the International Prospective Register of Systematic Reviews (PROSPERO) database [CRD420251001541].

Studies were included if they involved adult patients aged over 18 years with a diagnosis of HF based on established clinical or administrative criteria, directly compared cancer incidence between HF patients and non-HF controls, reported the incidence of any cancer diagnosed after HF, and provided relevant incidence data from observational study designs, including cohort or case–control studies published in English. Studies were excluded if they focused exclusively on paediatric populations, involved animal research or in vitro experiments, failed to establish a clear temporal relationship between HF diagnosis and subsequent cancer incidence, lacked a non-HF comparator group, or were review articles, case reports, editorials, or conference abstracts.

### 2.2. Definitions and Study Outcomes

HF was defined according to validated methods employed in the included studies. In registry-based and administrative database studies, HF was identified using International Classification of Diseases (ICD) diagnostic codes recorded during hospitalisations or outpatient encounters [2,3,4]. In clinically detailed cohort studies, HF was defined using guideline-based diagnostic frameworks that included objective assessments such as evaluations by cardiologists, echocardiography, and B-type natriuretic peptide (BNP) levels [12,13,14,15]. Instances of HF subtypes, HF with HFrEF or HF with HFpEF, were noted when available. Meanwhile, cancer was defined as an incident of malignancy diagnosed after HF, with most studies excluding non-melanoma skin cancer (NMSC). Diagnoses were confirmed through national cancer registries, hospital coding, or histopathological records.

The primary outcome of the systematic review is the cancer incidence, assessed through rates, proportions, or HRs as reported in each study included in the review. For the quantitative meta-analysis, the primary focus is on pooled HR for the incidence of all-site cancer in patients with HF compared to non-HF controls. Secondary outcomes included pooled HRs for specific cancer types, such as lung, colorectal, breast, prostate, haematological, and urinary tract cancers. Furthermore, a qualitative narrative synthesis was conducted for studies that were excluded from the meta-analysis due to the absence of a direct non-HF comparator or incompatible HRs.

### 2.3. Data Sources and Search Strategy

A comprehensive search of the medical literature published in English was conducted using four electronic databases, namely, PubMed, Web of Science, Scopus, and ScienceDirect, covering publications up to March 2025. Additionally, a manual search of the citations from the included articles was performed to identify further literature that may not have been captured in the initial search. Finally, a statistical meta-analysis was undertaken to synthesise and combine the results from the selected studies [16,17]. A systematic literature search was performed using MeSH terms and free-text keywords for heart failure (“heart failure,” “preserved ejection fraction,” “HFpEF,” “reduced ejection fraction”) and cancer incidence (“incidence of cancer”). An example search string was: (“incidence of cancer” [Title/Abstract]) AND ((“heart failure”) OR (“preserved ejection fraction”) OR (“HFpEF”) OR (“reduced ejection fraction”)). The full search strategy is detailed in Supplementary File S1.

In the literature search, two authors (BK and DA) independently screened the titles and abstracts of all articles. Any disagreement between the two authors was resolved by discussion with another author (SM). The selected articles were downloaded from the database, and the full texts of the relevant papers were examined to evaluate their eligibility for inclusion.

### 2.4. Data Extraction

Data from the included studies were extracted independently by two authors (LS and RS), with accuracy subsequently verified by a third author (SM). The following information was extracted: (1) study characteristics: author, publication year, country, study design and duration, sample size, country; (2) participant demographics, including age, gender, ethnicity, and comorbidities; (3) details of HF characteristics, including definition, subtypes (HfpEF or HFrEF), duration, severity, follow-up duration (years ± SD), and time to cancer diagnosis post-HF; (4) risk factors, including hypertension, dyslipidaemia, diabetes mellitus, myocardial infarction, and other relevant risk factors; (5) cancer outcomes, including subgroup analysis of the primary outcome (cancer incidence), secondary outcome (subtypes of cancers), type, time to cancer diagnosis, and post-HF incidence rates; (6) HR, including adjusted HR for age, gender, risk factors, lifestyle variables, and treatments received; (7) other factors: lifestyle variables, treatments received, and study quality (Supplementary File S2). Disagreements between reviewers during data extraction were resolved through discussion, with a third reviewer consulted if necessary.

### 2.5. Quality Assessment

A quality assessment of the included studies was conducted by one researcher (NA) to identify their risk of bias, utilising the Newcastle–Ottawa Scale (NOS) for cohort and case–control designs [18]. The NOS is a quality scale that assesses studies according to three main categories: selection (up to four stars), comparability (up to two stars), and exposure/outcome (up to three stars). This scale has a total score of 9 stars, and a study with scores between 7 and 9 is regarded as having “high quality”, while those with lower ratings were categorized as “low quality” [19]. Discrepancies in opinion during the evaluation were addressed through collaborative discussion or by consulting a third researcher. The quality score of the articles following this assessment is provided in Supplementary File S3.

### 2.6. Data Analysis

A qualitative narrative synthesis was performed on all included studies. For studies that were deemed eligible for quantitative meta-analysis (i.e., reported the cohort design, non-HF control group, cancer incidence outcome, and reporting of an HR with 95% confidence interval [CI]), the natural algorithm of their HR (log HR) and standard error (SE) were calculated. When the HR and its 95% CIs were reported, the SE was derived as:

Standard Error (SE) = [ln(upper CI) − ln(lower CI)]/3.92

where 3.92 is the two-sided z value for a 95% CIs (1.96 × 2).

The meta-analysis was performed using Cochrane Review Manager (RevMan) version 5.4.1 (The Cochrane Collaboration, London, UK) [20]. A random-effects model with inverse variance weighting was used to pool the log HRs, as heterogeneity was expected due to differences in study populations, adjustment variables, and cancer definitions. The included studies reported both conventional HRs derived from Cox proportional hazards models and sub-distribution hazard ratios (sHRs) derived from Fine–Grey competing-risk models. These measures are not directly equivalent. Cause-specific HRs estimate the instantaneous event rate among individuals who remain event-free, whereas sHRs quantify the effect of covariates on the cumulative incidence of an event while accounting for competing risks [21,22]. In older HF populations, where mortality represents an important competing event, these approaches address related but distinct research questions [21,22]. Because only three studies were eligible for quantitative synthesis, subgroup analysis according to effect measure was not feasible. Therefore, both measures were pooled in the primary analysis to maximise statistical power, while acknowledging that this approach may introduce methodological heterogeneity and limit direct comparability of effect estimates [21,22].

Heterogeneity was assessed using the I^2^ statistic and the Cochran Q test. I^2^ values of 25%, 50%, and 75% were considered indicative of low, moderate, and high heterogeneity, respectively [23]. A *p*-value < 0.10 for the Q test was considered indicative of significant heterogeneity.

### 2.7. Sensitivity Analysis

A leave-one-out sensitivity analysis was conducted to evaluate the influence of each study on the pooled estimate. The meta-analysis was repeated iteratively, excluding one study at a time. This approach identifies whether any single study disproportionately drives the overall result. The exclusion of Mirabel (2025) in the leave-one-out analysis also functioned as a sensitivity analysis to evaluate the effects of combining conventional HRs and sHRs, given that this was the only study reporting sHRs [3].

The justification for not performing additional sensitivity analyses (e.g., by adjustment quality, study design, or cancer definition) in this study is that there were only three studies eligible for the meta-analysis. Further stratification would have resulted in subgroups with one or zero studies, which would lack statistical power and meaningful interpretation. Therefore, no additional sensitivity analyses were conducted beyond leave-one-out.

## 3. Results

### 3.1. Literature Search Results

The initial database search identified 244 publications: 46 from PubMed, 51 from Web of Science, 46 from Scopus, and 101 from ScienceDirect. After removing 73 duplicates, 171 articles were screened based on their titles and abstracts. Of these, 164 articles were excluded for various reasons: 31 were excluded due to their article type (e.g., systematic reviews, review articles, and conference abstracts), one was an animal study, and 132 were deemed irrelevant to the research question. Consequently, seven studies met the pre-specified eligibility criteria for the systematic review.

**Figure 1:**
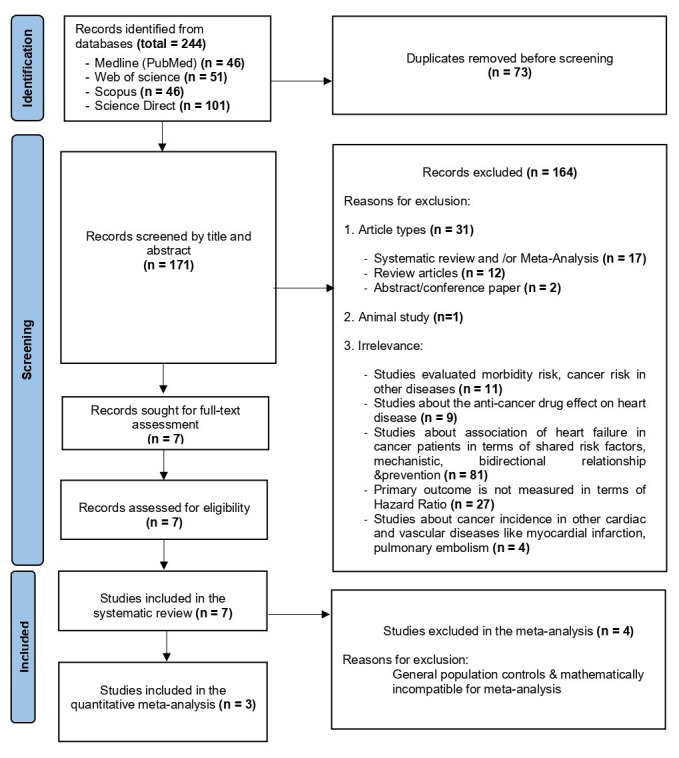
Flowchart and results of the literature search following the PRISMA 2020 guideline.

All seven studies were included in the qualitative narrative synthesis. Among these, three studies [3,4,12] directly compared cancer incidence between patients with HF and non-HF controls, providing HR with 95% CIs; therefore, these studies were included in the quantitative meta-analysis. The remaining four studies were part of the systematic review but were excluded from the meta-analysis for the following reasons: Bruhn et al. (2023) presented a temporal comparison (2016 vs. 1997) rather than a direct HF versus non-HF comparison [2]; Sagastagoitia-Fornie et al. (2022) [14] and Sakamoto et al. (2017) [13] compared HF patients to general population statistics; and Yoshihisa et al. (2019) [15] did not include a non-HF comparator. The research selection process is shown in the PRISMA flow diagram (Figure 1).

### 3.2. Characteristics of Included Studies

A total of seven studies, encompassing over 1.6 million participants (including HF patients and non-HF controls where reported), met the eligibility criteria for this systematic review, with data collection periods spanning from 2000 to 2019. These studies were conducted in Denmark [2,12], France [3], Germany [4], Spain [14], and Japan [13,15]. The study designs included retrospective registry-based cohorts [2,3,4,12,13], a prospective cohort [15], and a single-centre observational cohort [14]. Sample sizes for HF varied significantly, ranging from 1909 [14] to 330,867 [3]. Three studies included matched non-HF controls [2,3,4]; two compared HF patients with general population data [13]; and one study stratified HF patients based on prior cancer history without including a non-HF comparator [15]. Data collection periods varied from 4 to 19 years [2,4]. The main characteristics of the included studies are summarized in Table 1.

### 3.3. Definitions of Heart Failure and Cancer

The definition of HF varied across the included studies, influenced by study design and data source, yet all consistently relied on validated approaches. In large registry-based and administrative database studies, HF was identified using ICD diagnostic codes recorded during hospitalisations or outpatient encounters, reflecting clinician-diagnosed HF in routine clinical practice [2,3,4]. Mirabel et al. (2026) reported that 52.4% of HF patients had IHD [3]. Conversely, clinically detailed cohort studies employed guideline-based diagnostic frameworks bolstered by objective assessments. For example, Banke et al. (2016) defined HF through clinical evaluation and echocardiography conducted by an experienced cardiologist, with 89.3% of patients exhibiting left ventricular ejection fraction (LVEF) <45% [12].

Sakamoto et al. (2017) defined chronic HF using the Framingham Heart Failure Diagnostic Criteria, which was confirmed by cardiologist evaluation and supported by echocardiography and B-type natriuretic peptide levels [13]. Sagastagoitia-Fornie et al. (2022) and Yoshihisa et al. (2019) defined HF according to established clinical criteria in line with contemporary guidelines, incorporating imaging findings and clinical evaluations in hospitalised or cohort populations [14,15]. All studies utilised validated diagnostic strategies, whether through administrative coding or clinically adjudicated criteria, despite methodological heterogeneity, thereby ensuring a reliable identification of HF across diverse settings.

Yoshihisa et al. (2019) categorised HF into two types: HF with HFrEF and HF with HFpEF [15]. They reported that a history of cancer was associated with an increased risk of all-cause mortality in patients with HFrEF (*p* < 0.001) and a higher incidence of cardiac events in those with HFpEF (*p* = 0.011) [15]. However, disease severity assessments, including the New York Heart Association (NYHA) functional class, were not consistently reported across the included studies and could not be extracted from the available data.

Cancer was defined as a newly diagnosed malignancy that developed after the onset of HF. Most studies excluded NMSC from the primary outcome. Cancer diagnoses were verified through linkages to national cancer registries [2,3,4], hospital discharge codes [12], or clinical records that included histopathological confirmation [13,14,15]. Site-specific cancers were categorised using ICD codes or standard anatomical classifications, which encompassed lung, colorectal, breast, prostate, haematological (blood/lymphoid), and urinary tract malignancies.

### 3.4. Baseline Demographics and Comorbidities

The baseline characteristics of HF patients differed across the studies, affected by geographical location and cohort size. The mean age of the patients ranged from 64.4 years [14] to 77.7 years [3]. Intermediate mean ages were reported as 64.0 years [13], 67.8 years [12], and 72.6 years [4], while a median age of 68.7 years was noted by Bruhn et al. (2023) [2]. Yoshihisa et al. (2019) reported mean ages of 73.3 years for patients with a history of cancer and 66.1 years for those without such a history [15]. The proportion of female patients ranged from 4.1% [13] to 54.7% [3].

Hypertension was identified as the most prevalent risk factor, affecting up to 89.2% of patients [3]. The prevalence of diabetes ranged from 15% [12] to 41.5% among individuals with a history of cancer [15]. The occurrence of ischaemic heart disease (IHD) was reported between 22% and 54% [2,3,12,14]. Atrial fibrillation (AF) was reported in 24% to 44% of HF patients [2,12,14,15]. The prevalence of chronic obstructive pulmonary disease (COPD) ranged from 10% to 30% [2,14,15]. Dyslipidemia was reported solely by Sagastagoitia Fornie et al. (2022) (53–56%) and Yoshihisa et al. (2019) (70–72%) [14,15]. Chronic kidney disease (CKD) and anaemia were exclusively reported by Yoshihisa et al. (2019) (65% vs. 54% for CKD; 66% vs. 51% for anaemia, comparing those with and without a prior cancer history) [15]. Other comorbidities included obesity (16% according to Roderburg et al. (2021) [4], 4% for morbid obesity as noted by Mirabel et al. (2025) [3], valvular heart disease (39%), and dilated cardiomyopathy (12%) as reported by Sakamoto et al. (2017) [13].

The control groups used varied: Mirabel et al. and Roderburg et al. [3,4] employed matched non-HF controls; Sakamoto et al. (2017) and Sagastagoitia Fornie et al. (2022) [13,14] compared their findings to general population data; Yoshihisa et al. stratified HF patients based on prior cancer history without including an HF comparator [15].

### 3.5. Cancer Incidence of HF Versus Non-HF Groups

**Figure 2:**
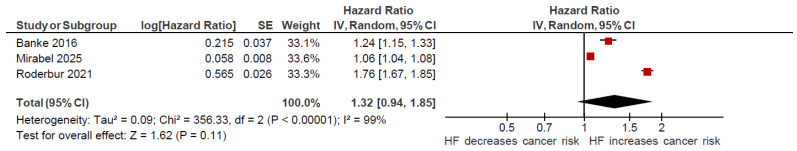
Forest plot of the association between pre-existing heart failure and cancer incidence [3,4,12]. Squares stand for study-specific hazard ratios (HRs) with 95% confidence interval (CI); square size is proportional to inverse variance weight. The diamond shows the pooled random effects of HR (1.32, 95% CIs 0.94–1.85). Heterogeneity: I^2^ = 99% (*p* < 0.00001). SE = standard Error; IV = inverse variance; HF = heart failure.

In a random-effects meta-analysis involving three cohort studies [3,4,12] (*n* = 440,298 patients with HF and 1,092,725 non-HF controls), pre-existing HF was not found to be associated with a significant increase in the incidence of cancer (pooled HR 1.32, 95% CIs 0.94–1.85; *p* = 0.11) (Figure 2). Individual study contributions were as follows: Banke (2016): HR 1.24 (1.15–1.33) indicates moderate risk with minimal adjustment (age and sex only) [12]; Mirabel (2025): HR 1.06 (1.04–1.08) reflects a small but precise risk, with the most comprehensive adjustment [3]; and Roderburg (2021): HR 1.76 (1.67–1.85) indicates the highest risk, with minimal adjustment and the addition of NMSC [4]. The forest plot shows that Mirabel’s estimate is among the most exact, as shown by its very narrow CI. This level of accuracy enhances the significance of the pooled effect, which typically results in the summary estimate shifting closer to the null. Conversely, Roderburg’s estimate suggests a positive association [4].

Considerable heterogeneity was observed (I^2^ = 99%, *p* < 0.00001), and the wide CI, which crosses the null value, prevents a definitive conclusion. The causes of heterogeneity are likely attributable to variations in adjustment strategies and outcome definitions across the three studies. Mirabel et al. conducted multivariable adjustments for lifestyle factors, including smoking, alcohol use, and obesity, as well as comorbidities, resulting in an HR close to 1.0 [3]. In comparison, Roderburg et al. relied mainly on matching for variables such as age, sex, diabetes, obesity, and consultation frequency, without employing multivariate adjustment [4].

This approach led to the highest HR at 1.76. Furthermore, Roderburg included NMSC in their cancer outcome measures (ICD-10 C00-C99, with skin tumours reported separately; HR 1.83) [4]. NMSC is particularly prevalent in older populations and may have overestimated the overall HR. Additional heterogeneity may arise from differences in population characteristics, such as age distribution and baseline cancer risk, as well as variations in healthcare systems across Denmark, France, and Germany. These methodological and demographic differences prevent a straightforward pooled estimate and highlight the necessity for standardised confounder adjustments and the exclusion of NMSC in future studies.

### 3.6. Narrative Synthesis of Included Studies

Although the risk magnitude varied across the papers, all seven studies consistently demonstrated that patients with HF had a higher cancer incidence than non-HF or general population controls. This narrative synthesis is structured into three key areas: studies included in the quantitative meta-analysis (*n* = 3), studies evaluated exclusively in the narrative synthesis (*n* = 4), and the consistency of the association across the literature.

#### 3.6.1. Studies Included in the Quantitative Meta-Analysis

Three studies directly compared cancer incidence between patients with HF and non-HF controls, and these were included in the quantitative meta-analysis [3,4,12]. Banke et al. (2016) reported a cancer incidence of 10.5% (975/9307) in Danish HF outpatients over a median follow-up of 4.5 years, with an adjusted HR of 1.24 (95% CIs: 1.15–1.33) after controlling for age and sex [12]. In the French nationwide cohort conducted by Mirabel et al. (2025), 8.5% (28,151/330,867) of HF patients developed cancer, resulting in an adjusted sub-distribution HR of 1.06 (95% CIs: 1.04–1.07; *p* < 0.0001) [3]. Roderburg et al. (2021) noted the largest absolute difference, with a cancer incidence of 25.7% in the HF cohort compared to 16.2% in matched non-HF controls, corresponding to an HR of 1.76 (95% CIs: 1.71–1.81; *p* < 0.001) [4]. Sex-stratified analyses revealed HRs of 1.85 (95% CIs: 1.77–1.92) for women and 1.69 (95% CIs: 1.63–1.76) for men (both *p*< 0.001) [4].

#### 3.6.2. Studies Included Exclusively in the Narrative Synthesis

Four studies were included only in the qualitative narrative synthesis because they lacked a direct non-HF control group, used general population comparators, or reported incompatible outcome metrics [2,13,14,15]. Bruhn et al. (2023) reported that the 5-year cancer risk in Danish patients with HF remained stable at approximately 9.0% from 1997 to 2016 [2]. When comparing the data from 2016 to that of 1997, the HR was 1.09 (95% CIs: 0.97–1.23), which was not statistically significant [2]. The most common cancer subtypes were gastrointestinal, pulmonary, and breast [2]. Sagastagoitia Fornie et al. (2022) reported 165 new cases of cancer (excluding NMSC among 1909 patients with HF over a median follow-up period of 4.07 years, resulting in an incidence rate of 1872 cases per 100,000 patient-years [14].

The most frequent cancer subtypes were lung (23.6%), colorectal (11.5%), prostate (10.9%), lymphoma (9.7%), breast (6.1%), and bladder (5.5%). The study compared HF patients to general population statistics rather than a concurrent non-HF control group [14]. Sakamoto et al. (2017) found a crude cancer prevalence of 3.78% (198 out of 5238) in the HF cohort, in contrast to a prevalence of 0.59% (749,767 out of 127,692,000) in the general Japanese population [13]. After age- and sex-standardisation, the prevalence was 2.27% (95% CI: 1.89–2.71) in the HF cohort versus 0.59% in controls, yielding a prevalence ratio of 3.85 [13]. Site-specific cancers showed substantially higher prevalence in HF patients: stomach (9× higher), breast (8.8×), prostate (8×), lung (5.0×), colon (4.2×), and other cancers (5.6×) [13].

Finally, Yoshihisa et al. (2019) [15] found that new cancer diagnoses after hospitalisation for HF occurred in 6.2% of patients, with identical proportions in those with prior cancer (17/275, 6.2%) and those without (114/1828, 6.2%). It is worth noting that this study did not include a non-HF control group but concentrated on cancer incidence and outcomes within the HF population [15]. Among patients with prior cancer, the most frequent cancer types were stomach, colorectal, blood/lymph, prostate, breast, and lung. Among patients without prior cancer, new post-HF cancers included stomach, colorectum, lung, blood/lymph, and liver [15].

#### 3.6.3. Consistency of Association Across Studies

Despite the methodological heterogeneity, all seven studies exhibited a consistent direction of effect, indicating an elevated incidence or prevalence of cancer among patients with HF. Among the three studies that reported HR, the magnitude of the association varied significantly, ranging from a modest 6% increase in incidence risk (sHR 1.06) [3] to a substantial 76% increase in incidence risk (HR 1.76) [4]. Furthermore, in the two studies that used general population comparators, cancer prevalence was consistently found to be higher in HF patients, with Sakamoto et al. (2017) reporting a prevalence ratio of 3.85 (standardised prevalence: 2.27% compared to 0.59%) [13].

### 3.7. Influence of Individual Studies on the Pooled Estimate

A leave-one-out sensitivity analysis demonstrated that the pooled HR varied when individual studies were omitted, ranging from 1.14 (95% CIs 0.98–1.33) following the exclusion of Roderburg (2021) [4] to 1.48 (95% CIs 1.05–2.08) after omitting Mirabel (2025) [3]. The overall finding became statistically significant only upon the removal of Mirabel (2025) [3], suggesting that this study, being the largest and most comprehensively adjusted, has a considerable influence on the pooled estimate (Supplementary File S4). Given the limited number of studies included, these results should be interpreted with caution.

### 3.8. Subgroup Analyses of Specific Cancer Types

**Figure 3:**
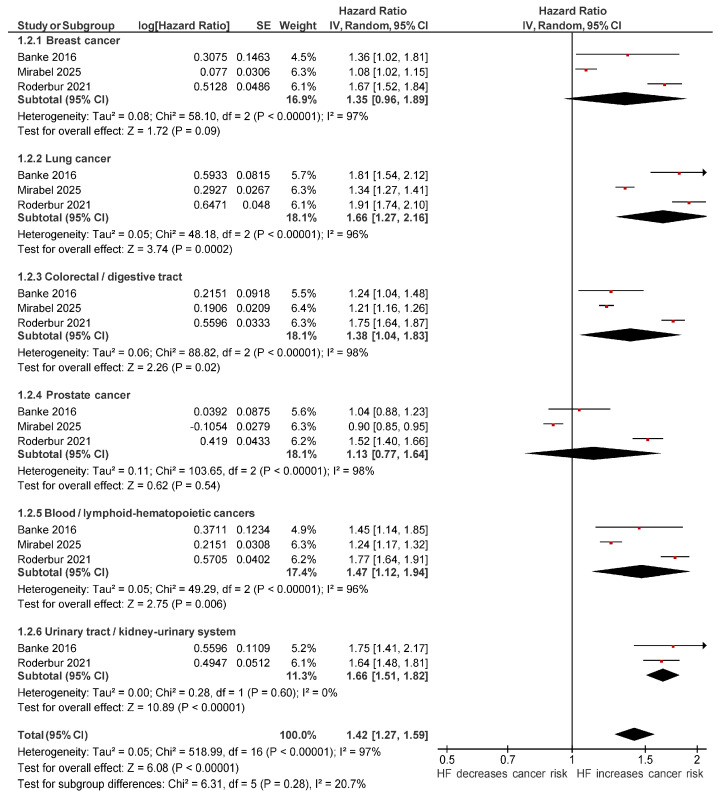
Forest plot of site-specific cancer risks in patients with pre-existing heart failure [3,4,12]. SE = standard error; IV = inverse variance; HF = heart failure.

Subgroup analyses were conducted for six cancer types (Table 2), as illustrated in the forest plot (Figure 3). In random-effects meta-analyses of three cohort studies, pre-existing HF was linked to a significantly elevated risk of lung cancer (pooled HR 1.66, 95% CIs 1.27–2.16; *p* = 0.0002), urinary tract/kidney cancer (HR 1.66, 95% CIs 1.51–1.82; *p* < 0.00001), blood/lymphoid-haematopoietic cancers (HR 1.47, 95% CIs 1.12–1.94; *p* = 0.006), and colorectal/digestive tract cancer (HR 1.38, 95% CIs 1.04–1.83; *p* = 0.02). Nevertheless, the risks associated with breast cancer (HR 1.35, 95% CIs 0.96–1.89; *p* = 0.09) and prostate cancer (HR 1.13, 95% CIs 0.77–1.64; *p* = 0.54) were not significant.

Most cancer sites exhibited high heterogeneity (I^2^ = 96–98%), apart from urinary tract cancer, where no heterogeneity was detected (I^2^ = 0%, based on only two studies). The overall pooled estimate presented at the bottom of Figure 2 (HR 1.42, 95% CIs 1.27–1.59) aggregates all site-specific analyses and should not be interpreted as the overall cancer risk. In contrast, the primary meta-analysis for any cancer reported a pooled HR of 1.32 (95% CIs 0.94–1.85).

In the above forest plot, squares represent study HRs, with size proportional to weight, while diamonds indicate pooled HRs with 95% CI. Significant increases were observed for lung, urinary tract/kidney, blood/lymphoid, and colorectal cancers. In contrast, breast and prostate cancers did not show significant associations. High heterogeneity was noted for most sites (I^2^ = 96–98%) apart from urinary tract cancers, which exhibited full consistency (I^2^ = 0%). The overall pooled estimate at the bottom (HR 1.42, 95% CIs 1.27–1.59) is a summary figure derived from all site-specific analyses; however, it does not accurately represent the overall cancer risk (see primary analysis: HR 1.32, 95% CIs 0.94–1.85).

### 3.9. Sensitivity Analyses

Sensitivity analyses showed that the exclusion of the studies by Banke et al. (2016) [12] or Roderburg et al. (2021) [4] did not materially affect the pooled estimate. In contrast, the exclusion of Mirabel et al. (2025) [3], which reported a sub-distribution hazard ratio, resulted in a higher pooled effect estimate that achieved statistical significance (HR = 1.48, 95% CI: 1.05–2.08; *p* = 0.03) compared with the primary analysis (HR = 1.32, 95% CI: 0.94–1.85). Nevertheless, this result should be interpreted cautiously given that only two studies contributed to the sensitivity analysis.

## 4. Discussion

### 4.1. Heart Failure and Cancer Risk

This systematic review and meta-analysis synthesised data from seven studies involving over 1.6 million participants to evaluate the association between HF and cancer risk. The pooled analysis of three studies [3,4,12] produced an HR of 1.32 (95% CIs: 0.94–1.85; *p* = 0.11), suggesting a 32% increased risk of cancer in HF patients compared to non-HF controls; however, this finding did not achieve statistical significance. Significant associations were shown for specific cancer sites, including lung, urinary tract, haematological, and colorectal cancers. All seven studies included in the analysis consistently showed a higher incidence or prevalence of cancer among patients with HF, despite notable variability in their methodologies. Our findings are broadly consistent with a recent meta-analysis by Jaiswal et al. (2023), which included 9 studies with 7,329,706 participants and reported a pooled HR of 1.43 (95% CIs: 1.21–1.68; *p* < 0.001) for cancer incidence in HF patients [6], with our slightly lower estimate of 1.32 likely reflecting differences in included studies, follow-up durations, and adjustment covariates. A key contributor to the substantial heterogeneity observed across studies included in the meta-analysis is the significant variation in confounder adjustment. Mirabel et al. (2025) conducted the most thorough adjustment, accounting for factors such as age, sex, cardiovascular comorbidities, region, and lifestyle elements including tobacco use, alcohol consumption, and obesity, which resulted in a sub-distribution HR of 1.06, the smallest effect estimates among the three studies [3]. In contrast, Roderburg et al. (2021) relied solely on matching for age, sex, diabetes, obesity, and consultation frequency, without any multivariable adjustment, yielding the highest HR of 1.76 [4]. Banke et al. (2016) fell in between, adjusting only for age and sex, resulting in an HR of 1.24 [12]. This gradient strongly indicates that residual confounding, particularly from smoking, alcohol, and socioeconomic status, may significantly inflate the observed association in studies with inadequate adjustment.

Smoking is a well-recognised risk factor for both HF and various types of cancer, including lung, colorectal, and haematological malignancies [24]. Additionally, factors such as alcohol consumption, obesity, physical inactivity, and socioeconomic status represent shared risk factors that could potentially confound the relationship between HF and cancer if not adequately addressed [24]. It is crucial to note that even the most comprehensively adjusted observational studies cannot eliminate residual confounding. This is because aspects such as detailed smoking history, exposure to passive smoke, dietary patterns, levels of physical activity, and socioeconomic status may still be imperfectly measured or entirely unmeasured [25].

The inclusion of NMSC in Roderburg’s outcome definition may have inflated its HR, because this type of malignancy is predominantly linked to sun exposure and age, rather than HF related pathways [4,24]. Observations suggest that the true independent effect of HF on cancer risk, if it exists, is likely modest, and much of the reported excess risk in earlier studies may occur from insufficient adjustment for shared risk factors. Future studies should implement standardised and comprehensive adjustment sets that encompass smoking (with detailed quantification), alcohol consumption, obesity, physical activity, and socioeconomic indicators to reduce residual confounding and facilitate more meaningful comparisons across populations.

The pooling of conventional HRs and sHRs warrants cautious interpretation because these effect measures arise from different competing-risk frameworks and estimate different quantities [21,22]. Although pooling was necessary owing to the limited number of eligible studies, the sensitivity analysis excluding Mirabel et al. (2025) [3] yielded a higher pooled estimate (HR = 1.48, 95% CI: 1.05–2.08; *p* = 0.03) than the primary analysis (HR = 1.32, 95% CI: 0.94–1.85). This finding suggests that inclusion of the competing-risk estimate may have attenuated the observed association between HF and cancer incidence. The observed difference may be attributed to the methodological characteristics of competing-risk models, which account for competing events and could yield more conservative estimates in populations facing significant non-cancer mortality. Nevertheless, the limited number of studies included and the high heterogeneity hinder definitive conclusions about the effect measure’s influence on the pooled estimate. Future research that employs standardised analytical approaches and consistent effect measures would improve comparability and support a more robust synthesis of evidence.

For site-specific cancers, Jaiswal et al. (2023) reported a statistically significant association with breast cancer (HR 1.28; 95% CIs: 1.09–1.50) [6], whereas our estimate did not reach statistical significance (HR 1.35; 95% CIs: 0.96–1.89; *p* = 0.09). Both meta-analyses identified lung cancer as the highest-risk malignancy. Jaiswal et al. (2023) reported an HR of 1.89 (95% CIs: 1.25–2.85) [6]. In comparison, our HR of 1.66 was directionally consistent with the estimate reported by Banke et al. (2016), which is included in the analysis and found an 81% elevated risk (HR 1.81; 95% CIs: 1.54–2.12) [12]. This association may be due to shared risk factors, particularly smoking, prevalent in both conditions. Furthermore, persistent inflammation and oxidative stress caused by HF may lead to carcinogenesis in vulnerable tissues, such as the lungs [26,27].

Colorectal cancer estimates were remarkably similar between our study and Jaiswal et al.’s meta-analysis (HR 1.38, 95% CIs 1.04–1.83; *p* = 0.02 vs. HR 1.32, 95% CIs 1.11–1.57; *p* < 0.001, respectively), and neither analysis identified a significant association with prostate cancer (HR 1.13, 95% CIs: 0.77–1.64; *p* = 0.54 vs. HR 0.97, 95% CIs 0.66–1.43; *p* = 0.88, respectively). The higher incidence of colon cancer in HF patients in our study is consistent with the results from Mirabel et al. (2025), which reported a 21% increased risk (1.21; CI 1.16–1.26; *p* < 0.0001) [3]. The association can be linked to chronic gut hypoperfusion in HF, which results in intestinal barrier dysfunction, dysbiosis, and chronic inflammation elements recognised as contributors to colorectal carcinogenesis [28,29]. Our observed risk for haematological malignancies (HR 1.47, 95% CIs: 1.12–1.94; *p* = 0.006) is consistent with the findings of Jaiswal et al. (2023), who reported an HR of 1.63 (95% CIs: 1.15–2.33; *p* = 0.01) [6]. Both studies suggest a significantly increased risk among patients with HF. A novel finding from our study was the significant association with urinary tract cancers (HR 1.66; 95% CIs: 1.51–1.82; *p* < 0.00001), which showed no heterogeneity (I^2^ = 0%), thereby providing the most reliable estimate.

This finding warrants further investigation. It may be linked to CKD, which often coexists with HF and has been associated with an increased risk of cancer [30]. Both meta-analyses noted significant heterogeneity across many cancer types (I^2^ = 96–98% in this meta-analysis versus I^2^ = 59.32–99.30% in the Jaiswal et al. meta-analysis) and identified diabetes mellitus as a potential positive effect modifier [6]. The significant heterogeneity observed in our study, excluding urinary tract cancers, is likely attributable to several factors. These include variations in the definitions of HF, the absence of stratification between HFrEF and HFpEF, and differing follow-up durations that range from 4 to 19 years. Additionally, inconsistent adjustments for confounding factors such as smoking, regional disparities in screening practices, and variations in medication exposure further contribute to this heterogeneity, which may lead to differing outcomes in HF patients across various demographics and treatment protocols. Research indicates that follow-up duration, age, and the prevalence of hypertension significantly account for the heterogeneity observed between studies [30].

The lack of HF phenotype stratification is particularly important, as patients with HFrEF and HFpEF show differing cancer-related outcomes [31]. Inconsistent adjustments for confounders can significantly impact effect estimates. For example, the odds ratios for the association between HF and cancer decreased from 3.23 when unadjusted to 1.33 following the adjustments for demographic and clinical variables [32]. This variation highlights the need for standardised definitions, consistent adjustments, and stratification by HF phenotype in future research.

The quality assessment conducted using the Newcastle–Ottawa scale indicated that the studies included generally exhibited high methodological quality. However, substantial heterogeneity was observed across these studies (I^2^ = 96–98%), which reflects variations in study design, population demographics, the severity of HF, follow-up periods, and confounding variables. This highlights the complex and multifaceted relationship between HF and cancer, necessitating a cautious interpretation of the pooled estimates and underscoring the need for subgroup analyses to explore the underlying causes of this heterogeneity. Our findings corroborate and extend previous evidence suggesting a bi-directional relationship between cancer and cardiovascular disease [33,34]. Earlier systematic reviews predominantly concentrated on the cardiotoxic effects of cancer treatments, overlooking the inverse relationship examined in our study. Although comprehensive meta-analytic evidence is lacking, pioneering research by Koene et al. (2016) introduced the concept of “reverse cardio-oncology”, proposing that cardiovascular disease may increase susceptibility to cancer development [33].

A further consideration when interpreting the association between HF and cancer incidence is the potential for surveillance bias. Patients with HF generally have more frequent outpatient follow-up, hospital admissions, laboratory investigations, imaging studies, and specialist assessments, which may increase the likelihood of detecting previously unrecognised malignancies compared with individuals without HF. Consequently, part of the observed increase in cancer incidence may reflect enhanced diagnostic intensity rather than a true biological effect. Some included studies attempted to minimise this bias through adjustment for healthcare utilisation and major confounders; for example, Roderburg et al. matched participants for consultation frequency, whilst Mirabel et al. employed extensive multivariable adjustment for demographic, lifestyle, and clinical factors. However, residual surveillance bias cannot be entirely excluded and should be considered when interpreting the findings of this review. The persistence of increased risks for several site-specific cancers after adjustment suggests that both enhanced surveillance and shared pathophysiological mechanisms may contribute to the observed association [3,4]. Future prospective studies should incorporate measures of healthcare utilisation and diagnostic intensity, such as consultation frequency, hospital admissions, screening practices, and imaging exposure, to better distinguish true biological associations from surveillance-related effects.

Several molecular mechanisms may elucidate the observed association between HF and cancer incidence. First, chronic systemic inflammation, a hallmark of both HF and carcinogenesis, likely plays a critical role. In HF, elevated levels of interleukin 6 (IL 6), C-reactive protein, and tumour necrosis factor alpha (TNF α) may facilitate oncogenic processes by promoting tumour initiation and growth [35,36]. These inflammatory mediators can inflict DNA damage, inhibit apoptosis, promote angiogenesis, and create a microenvironment conducive to cancer cells [37,38]. Second, neurohormonal activation in HF, particularly the sustained elevation of catecholamines and the activation of the RAAS, may promote carcinogenesis [39,40]. Beta adrenergic signalling has been shown to enhance tumorigenesis, metastasis, and angiogenesis in preclinical models [41,42].

Third, tissue hypoxia resulting from reduced cardiac output may trigger hypoxia-inducible factors, which regulate genes involved in angiogenesis, cellular proliferation, and metabolic reprogramming processes essential to carcinogenesis [43]. Fourth, oxidative stress significantly increased in HF due to mitochondrial dysfunction, NADPH oxidase activation, and reduced antioxidants; it can cause DNA damage and genomic instability, which are key initiating molecular events in carcinogenesis [44,45]. Additionally, shared risk factors such as obesity, diabetes, smoking, and physical inactivity exacerbate the association between HF and cancer by promoting both conditions through common pathophysiological mechanisms, including insulin resistance and chronic inflammation [46,47].

The aetiologies of cardiac arrhythmias, systemic hypertension, and ischaemic heart disease have significant mechanistic overlaps with the pathophysiological cascade leading to HF. Persistent tachyarrhythmias like atrial fibrillation and pathological QT or QRS interval prolongation are examples of chronic electrophysiological disturbances that often cause detrimental electrical, metabolic, and structural remodelling that upsets calcium homeostasis and causes arrhythmia-induced cardiomyopathy [48]. At the same time, persistent systemic hypertension induces a chronic mechanical afterload that leads to a unique phenotypic spectrum. This maladaptive remodelling usually begins as compensatory left ventricular hypertrophy (LVH), gradually progresses to the diastolic dysfunction typical of HF with preserved ejection fraction (HFpEF), and can eventually degenerate into eccentric chamber dilatation and HF with reduced ejection fraction (HFrEF) [49]. Ischaemic cardiomyopathy, in which cardiac perfusion is compromised by macrovascular obstructive lesions and coronary microvascular dysfunction, often exacerbates this structural degeneration and causes permanent fibrotic scarring and chronic hibernation [49]. These crossing pathways are highlighted by strong clinical and translational evidence, which validates them as a cohesive network of progressive cardiovascular failure rather than discrete comorbidities.

Immune checkpoint inhibitors (ICIs) have been associated with severe myocarditis and HF, highlighting the bidirectional nature of cardio-oncology [50]. Moreover, pre-existing HF may lead clinicians to refrain from administering ICIs, thereby affecting cancer survival outcomes. To address ICI-induced cardiotoxicity in patients with pre-existing HF, three strategies are available: (i) temporarily or permanently discontinuing the ICI; (ii) initiating high-dose corticosteroids, such as methylprednisolone; and (iii) adhering to guideline-directed HF therapy [51]. Alternative immunosuppressants, including mycophenolate mofetil, abatacept, and Janus kinase (JAK) inhibitors, such as ruxolitinib, can be utilised in cases of steroid-resistant conditions. A multidisciplinary cardio-oncology team is also essential for managing the interplay between cancer treatment and cardiovascular risks.

Clinically, our findings indicate that healthcare providers caring for patients with HF must remain alert for the development of cancer, particularly in relation to the high-risk types identified in this study. It is essential to implement age-appropriate cancer screening protocols, which may include earlier or more frequent screening intervals [52,53].

### 4.2. Study Limitations

The most critical limitation of this review is that the findings are based on observational data, which are susceptible to residual confounding from unmeasured or inadequately adjusted factors, such as age, smoking, comorbidity burden, and access to healthcare. Surveillance bias may also occur, as patients with HF tend to undergo more frequent medical testing, leading to higher rates of cancer detection. This presents a significant alternative explanation for the observed association; therefore, causality cannot be inferred. Residual confounding remains a significant concern, as even the most comprehensively adjusted studies cannot account for all potential confounders, such as detailed smoking history, passive smoking, dietary patterns, physical activity, and socioeconomic status. The inability to adjust for these factors may have led to biased estimates, particularly in studies with minimal adjustments. This issue persists despite the thoroughness of the adjustments made, highlighting the challenges in achieving accurate estimates in research.

Another major limitation is that we were unable to differentiate between HFrEF and HFpEF since the majority of the included studies did not report cancer incidence stratified by ejection fraction. This is significant since the underlying biology, comorbidity profiles, and possible correlation with cancer risk of distinct HF symptoms may differ significantly. Therefore, rather than being a risk estimate specific to a particular phenotype, the pooled estimates in this review should be seen as representing HF as a broad clinical entity. To determine whether the observed association is more strongly influenced by one subtype than the other, future studies should record cancer outcomes for HFrEF and HFpEF separately.

Substantial heterogeneity was observed across most cancer types (I^2^ = 96–98%), which likely reflects differences in HF definitions, follow-up durations (4–19 years), adjustment covariates, regional screening practices, and differential medication exposure. The pooled estimate for overall cancer (HR 1.32; 95% CIs: 0.94–1.85) did not reach statistical significance, partly due to the limited number of studies (*n* = 3) that provided hazard ratios with direct non-HF comparators.

Pooling conventional HRs and sHRs may have introduced methodological heterogeneity because these measures differ in both interpretation and handling of competing events. Exclusion of the only study reporting sHRs resulted in a statistically significant pooled estimate, suggesting that the overall findings may be sensitive to the choice of effect measure. However, the limited number of eligible studies prevented formal subgroup analyses. Future meta-analyses with a larger evidence base should consider stratifying analyses according to effect measures to evaluate their potential influence on pooled estimates.

The review was limited to seven studies, of which four were excluded from the primary meta-analysis due to the absence of a direct non-HF control group or the reporting of outcome metrics that were not mathematically compatible (e.g., prevalence ratios, temporal comparisons). Inconsistencies in reporting among the selected studies, such as variations in data presentation (e.g., number of cases vs. percentages, prevalence vs. incidence), also posed challenges for data extraction and synthesis.

In addition, we further note that the power to detect true heterogeneity is limited when few studies are available; for that, we further explored the observed heterogeneity by performing sensitivity analyses and subgroup analyses. Meta-regression was also considered to explore potential sources of heterogeneity; however, with only three studies included in the primary analysis, such an approach would be underpowered and could yield unstable or spurious results. The Cochrane Collaboration advises against employing meta-regression when there are fewer than ten studies in a meta-analysis [48]. Furthermore, the inability to adjust for family history of cancer or for the utilisation of specific HF medications (e.g., renin–angiotensin–aldosterone system inhibitors and beta-blockers) may have introduced residual confounding, particularly regarding specific cancer subtypes (e.g., haematological: 1.38 vs. 1.47), which could affect the accuracy and reliability of our findings.

Another limitation in this paper that should be considered lies in the fact that there were only three studies used for conducting meta-analysis. This means that the presence of publication bias could not be checked through constructing a funnel plot or performing tests like Egger’s regression test because of the insufficient number of papers; fewer than ten studies make it impossible to obtain valid results.

### 4.3. Future Directions

Future research should prioritise age-appropriate cancer screening protocols for patients with HF, particularly for high-risk malignancies such as lung, urinary tract, haematological, and colorectal cancers. A multidisciplinary approach that integrates cardiovascular and oncological care, along with targeted management of shared risk factors including obesity, diabetes, smoking, and physical inactivity, may enhance patient outcomes and reduce cancer risk in HF patients [53].

The emerging association between HF and urinary tract cancers necessitates further investigation, as does the impact of HF pharmacotherapies, including RAAS inhibitors and beta-blockers, on cancer risk. Cancer has become a significant contributor to mortality among HF patients, and RAAS-targeting medications have shown promise in reducing the risk of certain cancers [54]. However, real-world evidence suggests that while RAAS inhibitors may increase the risk of lung cancer, they could potentially lower the risk of liver, biliary, and gastric cancers. Conversely, beta-blockers may inhibit tumour growth and improve survival in patients with triple-negative breast cancer, although their effects within HF populations remain inconsistent, partially due to surveillance bias [55]. These findings highlight the need for both mechanistic and clinical research. To achieve these objectives, large prospective cohort studies should be conducted using standardised HF diagnostic criteria, consistent adjustment for shared risk factors including smoking, body mass index, and physical activity, and comprehensive documentation of cancer screening histories, as recommended by recent large-scale investigations [24].

Mechanistic studies that explore chronic inflammation, neurohormonal activation, gut dysbiosis, and oxidative stress are essential to clarify the biological links between HF and specific cancer types [53]. Given the documented regional and demographic variations in the HF–cancer association [56] and the disparities in cardiovascular risk factor management across diverse racial and socioeconomic groups [57,58], future studies must include varied populations and geographic regions to bolster external validity. International collaborative efforts to pool individual participant data would facilitate more robust adjustments for confounders and enhance the precision of site-specific cancer estimates [59,60].

## 5. Conclusions

This systematic review and meta-analysis found that although the pooled estimate for overall cancer risk among patients with heart failure was elevated (HR 1.32, 95% CI 0.94–1.85), the association did not reach statistical significance and was accompanied by substantial heterogeneity. However, significantly increased risks were observed for several site-specific cancers, including lung, urinary tract, haematological, and colorectal malignancies. The association with urinary tract cancer, which demonstrated a pooled HR of 1.66 with low between-study heterogeneity (I^2^ = 0%), represents a potentially important finding that warrants further investigation. Due to the high heterogeneity observed in overall cancer risk, these results should be interpreted with caution and should not be considered evidence of a causal relationship.

## Figures and Tables

**Table 1:** Characteristics of the included studies.

Study (Author, Year)	Design/Location	Sample Size	Age (yrs)	Female (%)	Key Cancer Associations with HF
Banke et al. (2016) [12]	Denmark; Retrospective cohort (2002–2012)	9307 HF pts	67.8 ± 2.2	27%	↑ Risk of atrial fibrillation; cancer incidence not stratified by type
Mirabel et al. (2025) [3]	France; Nationwide cohort (2010–2019)	330,867 HF pts vs. 992,601 controls	77.7 ± 13.5	54.70%	HF linked to ↑ morbid obesity, ↑ renal failure; cancer risk elevated but not subtype-specific
Bruhn et al. (2023) [2]	Denmark; Nationwide cohort (1997–2016)	103,711 HF pts	68.7 (60.4–74.8)	35.90%	HF linked to ↑ atrial fibrillation and COPD; cancer incidence not stratified
Roderburg et al. (2021) [4]	Germany; Retrospective cohort (2000–2018)	100,124 HF vs. 100,124 controls	72.6 ± 12.2	54%	HF associated with ↑ obesity; cancer incidence not subtype-specific
Sagastagoitia-Fornie et al. (2022) [14]	Spain; Single-center observational (2010–2019)	1909 HF pts	64.4 (15.9–94.2)	28.10%	HF linked to ↑ COPD and atrial fibrillation; cancer incidence not subtype-specific
Sakamoto et al. (2017) [13]	Japan; Retrospective single-center (2001–2015)	5238 HF pts	64 ± 12	4.14%	HF associated with valvular heart disease, dilated cardiomyopathy, hypertrophic cardiomyopathy; cancer incidence not stratified
Yoshihisa et al. (2019) [15]	Japan; Prospective observational (2010–2016)	2103 HF pts	Prior-CA: 73.3 ± 11.3; Non-CA: 66.1 ± 14.8	40% vs. 38.5%	Direct comparison of HF patients with vs. without prior cancer; ↑ CKD, anemia, AF, COPD in prior-CA group

AF, atrial fibrillation; CA, cancer; CKD, chronic kidney disease; COPD, chronic obstructive pulmonary disease; HF, heart failure; vs., versus. Bruhn et al. (2023) [2] reported a temporal comparison (2016 vs. 1997) rather than a direct HF versus non-HF comparison; therefore, it was excluded from the quantitative meta-analysis. For Bruhn et al. (2023) [2], the non-HF group was matched to the HF group; therefore, age and sex were not reported separately for the non-HF group. Sagastagoitia-Fornie et al. (2022) [14] and Sakamoto et al. (2017) [13] compared HF patients to general population statistics rather than a concurrent non-HF control group; therefore, they were excluded from the quantitative meta-analysis. Yoshihisa et al. (2019) [15] lacked a non-HF comparator and stratified HF patients by prior cancer history; therefore, it was excluded from the quantitative meta-analysis.

**Table 2:** Summary of the association between heart failure and site-specific cancers.

Cancer Type	Contributing Studies	Pooled HR (95% CI)	*p*-Value	I^2^ (%)	Interpretation
Breast Cancer	Banke 2016 [12], Mirabel 2025 [3], Roderburg 2021 [4]	1.35 (0.96–1.89)	0.09	97	Trend toward increased risk but not statistically significant
Lung Cancer	Banke 2016 [12], Mirabel 2025 [3], Roderburg 2021 [4]	1.66 (1.27–2.16)	0.0002	96	Increased risk in HF patients
Colorectal/digestive tract Cancer	Banke 2016 [12], Mirabel 2025 [3], Roderburg 2021 [4]	1.38 (1.04–1.83)	0.02	98	Moderately increased risk
Prostate	Banke 2016 [12], Mirabel 2025 [3], Roderburg 2021 [4]	1.13 (0.77–1.64)	0.54	98	No clear association observed
Blood/lymphoid - hematopoietic Cancer	Banke 2016 [12], Mirabel 2025 [3], Roderburg 2021 [4]	1.47 (1.12–1.94)	0.006	96	Increased risk in HF patients
Urinary tract/kidney-urinary system Cancer	Banke 2016 [12], Roderburg 2021 [4] *	1.66 (1.51–1.82)	<0.00001	0	Strong and consistent increased risk

* Footnotes: Mirabel’s study did not address urinary tract cancer. Pooled HRs were calculated using random-effects models (inverse variance methods) with RevMan 5.4.1. Heterogeneity assessed with I^2^ statistic. HF, heart failure; HR, hazard ratio; CI, confidence interval.

## Data Availability

The original contributions presented in this study are included in the article/Supplementary Materials. Further inquiries can be directed to the corresponding author.

## Supplementary Materials

The following supporting information can be downloaded at: https://www.mdpi.com/article/10.3390/diagnostics16132016/s1, Supplementary File S1. Search strategy; Supplementary File S2. Data extraction sheet; Supplementary File S3. Quality assessment of the included studies; Supplementary File S4. Sensitivity analysis.

## Abbreviations

The following abbreviations are used in this manuscript:

ACEi	Angiotensin converting enzyme inhibitor
AF	Atrial fibrillation
BNP	B-type natriuretic peptide
CA	Cancer
CAD	Coronary artery disease
CI/CIs	Confidence interval
CKD	Chronic kidney disease
COPD	Chronic obstructive pulmonary disease
CVD	Cardiovascular disease
DCM	Dilated cardiomyopathy
DM	Diabetes mellitus
HCM	Hypertrophic cardiomyopathy
HF	Heart failure
HFpEF	Heart failure with preserved ejection fraction
HFrEF	Heart failure with reduced ejection fraction
HR/HRs	Hazard ratio
HTN	Hypertension
ICD	International Classification of Diseases
IHD	Ischaemic heart disease
IL 6	Interleukin 6
IQR	Interquartile range
IR	Incidence rate
LVDd	Left ventricular diastolic diameter
LVEF	Left ventricular ejection fraction
MI	Myocardial infarction
NMSC	Non-melanoma skin cancer
NOS	Newcastle–Ottawa scale
