# Supplementary material for: The Association Between Cancer Incidence and Heart Failure: A Systematic Review and Meta-Analysis
Source: Diagnostics (Basel). 2026 Jun 28;16(13):2016. doi: 10.3390/diagnostics16132016 (PMC13359713; doi:10.3390/diagnostics16132016)
Supplement: Supplementary file 1 [file diagnostics-16-02016-s001.zip › Supplementary File S1 (Search strategy) .pdf]

Pubmed

Date: 24.3.2025

No. of hits: 46

("incidence of cancer"[Title/Abstract]) AND (((("heart failure") OR ("preserved ejection fraction")) OR ("HFpEF")) OR ("reduced ejection fraction"))

Web of science

Date: 24.3.2025

No. of hits: 51

TS=("Incidence of cancer") AND (((TS=("heart failure")) OR TS=("preserved ejection fraction")) OR TS=("HFpEF")) OR TS=("reduced ejection fraction")

Scopus

Date: 24.3.2025

No. of hits: 46

TITLE-ABS ("heart failure" OR "preserved ejection fraction" OR hfpef OR "reduced ejection fraction") AND TITLE-ABS ("incidence of cancer")

Science direct

Date: 24.3.2025

No. of hits: 101

("incidence of cancer"[Title/Abstract]) AND (((("heart failure") OR ("preserved ejection fraction")) OR ("HFpEF")) OR ("reduced ejection fraction"))
