# Supplementary material for: The Association Between Cancer Incidence and Heart Failure: A Systematic Review and Meta-Analysis
Source: Diagnostics (Basel). 2026 Jun 28;16(13):2016. doi: 10.3390/diagnostics16132016 (PMC13359713; doi:10.3390/diagnostics16132016)
Supplement: Supplementary file 1 [file diagnostics-16-02016-s001.zip › Supplementary File S2 (Data Extraction Sheet).pdf]

			**Age (years)**		**Female (%)**		**Risk Factors**					**Subgroup analysis**	**Outcome**		**HR (95% CI)**	**p value**	**HR adjusted for (95 CI)**
**Author and Year of Publication**	**Study location, study design, and duration**	**Sample size (n)**	**HF**	**Non-HF**	**HF**	**Non-HF**	**Hyper-tension**	**Dys-lipidemia**	**DM**	**MI**	**Others**		**Primary outcome (Incidence of cancer) (No. %)**	**Secondary outcome (Subtypes of cancers) (Please indicate the subtype of cancer)**			
Banke et al. (2016)	Denmark; Retrospective cohort study using national Danish registries (2002–2012)	9,307 HF patients	Mean= 67.8 ±11	Not reported	27 %	Not reported	Not reported	Not reported	15%	22.4%	Atrial fibrillation: 23.6% (2,196),<br><br>Other arrhythmic disorders: (6.5%)	Age-stratified (<60, 60–69, ≥70 years)	10.5% (975/9307)	Higher HRs for lung (1.81), skin (1.84), kidney (1.75), liver (1.60), blood (1.45), colon (1.24), breast (1.36); not significant for prostate (HR 1.04). Lung, skin, kidney/urinary, liver/biliary, lymph/blood, colon/rectal, breast (women), prostate (men)	1.24 (1.15–1.33)<br>**Secondary Outcomes (Subtypes of Cancers with HRs):**<br>Lung cancer: HR 1.81 (1.54–2.12), p < 0.0001<br>Skin cancer: HR 1.84 (1.57–2.15), p < 0.0001<br>Kidney/urinary system: HR 1.75 (1.41–2.18), p < 0.0001<br>Liver/biliary system: HR 1.60 (1.20–2.13), p = 0.0015<br>Lymph/blood cancer: HR 1.45 (1.14–1.85), p = 0.0027<br>Colon/rectal cancer: HR 1.24 (1.04–1.49), p = 0.018	All p value for HR of cancer and all subtypes are <0.05 except prostate cancer (p =0.635	Adjusted for age and gender

															Breast cancer (women): HR 1.36 (1.02–1.81), p = 0.038<br>Prostate cancer (men): HR 1.04 (0.88–1.24), p = 0.635 (not significant)		
Mirabel et al. (2025)	France; Nationwide cohort study using the French SNDS database (2010–2019)	Total participants: 1,323,468<br><br>HF patients: 330,867<br><br>Matched controls of non-HF participants: 992,601	Mean= 77.7 ±13.5	Mean = 77.7 ± 13.5	54.7%	54.7%	HF: 89.2%<br><br>Control: 66.1%	Not reported	HF: 20.6%<br>Control: 16.3%	HF: 52.4<br><br>Control: 36.9%	Morbid Obesity with HF: 12,759 (3.9%),<br><br>Morbid Obesity with no HF: 10,936 (1.1%),<br><br>Renal failure requiring dialysis: In HF (1,939; 0.6%) and in no HF (2,722; 0.3%)	Matched for age and sex (3:1 ratio)	8.5% (28,151)	Colorectal, lung, breast (women), prostate (men), blood malignancies (multiple myeloma)	Adjusted sHR = 1.06, [1.04–1.07]; P < 0.0001.<br>Subtypes of Cancers with sHRs:<br>Colorectal cancer: Adjusted sHR 1.21 (1.16–1.26), p < 0.0001<br>Lung cancer: Adjusted sHR 1.34 (1.27–1.41), p < 0.0001<br>Breast cancer (women): Adjusted sHR 1.08 (1.02–1.15), p = 0.01<br>Prostate cancer (men): Adjusted sHR 0.90 (0.85–0.95), p < 0.0001 (lower risk)<br>Blood malignancies: Adjusted sHR 1.24 (1.17–1.32), p < 0.0001	Any cancer<0.0001, colorectal <0.0001, Lung <0.0001, Breast ~0.01, Prostate< 0.01, Blood <0.0001	Age, Gender, ischemic heart disease, hypertension, diabetes, year of diagnosis, region, tobacco use, alcohol use, and obesity

3. Bruhn et al. (2023)	Denmark; Nationwide cohort study (1997–2016)	HF: 103,711	Mean= 68.7 (60.4–74.8)	Matched group	35.9%	Not reported	Not reported	42.8%	17.8%	53.9%	Atrial fibrillation: 32,337 (31.2%),<br><br>Chronic obstructive pulmonary disease (COPD): 21,030 (20.3%)	by age (<60 vs ≥60) and presence of ischemic heart disease (IHD) (Yes, No). Also stratified by sex, calendar year, and cancer type	- 5-year cancer risk remained stable at 9.0% (1997–2016) despite improved HF survival.	The most common subtypes of cancer during follow up were gastrointestinal, pulmonary and breast cancer	HR (2016 vs 1997): 1.09 (95% CI: 0.97–1.23), not significant Cancer types (most common): Gastrointestinal, pulmonary, breast.	Non-Significant (p-value not reported)	Gender, year, IHD, COPD, loop diuretics, antidiabetic drugs
Roderburg et al. (2021)	Germany; Retrospective cohort study (Jan 2000–Dec 2018)	Total participants: 200,248<br><br>HF patients: 100,124<br><br>Non-HF participants: 100,124	Mean= 72.6 ±12.2	Mean = 72.6 ±12.2	54%	54%	Not reported	Not reported	37.4%	Not reported	Obesity: 15.9% (matched in both cohorts)	By Gender:<br>- Women: HF 28.6% vs. non-HF 18.8% (HR: 1.85, P < 0.001)<br>- Men: HF 23.2% vs. non-HF 13.8% (HR: 1.69, P < 0.001)	HF cohort: 25.7%<br>Non-HF cohort: 16.2%	Cancer sites (ICD-10 codes):<br>- Lip/oral cavity/pharynx (C00–C14)<br>- Respiratory organs (C30–C39)<br>- Female genital organs (C51–C58)<br>- Skin (C43–C44)<br>- Lymphoid/ hematopoietic (C81–C96)<br>- Digestive organs (C15–C26)<br>- Breast (C50)<br>- Urinary tract (C64–C68)<br>- Male genital organs (C60–C63)	**Total**: HR 1.76 (1.71–1.81; P < 0.001)<br>**Women**: HR 1.85 (1.77–1.92; P < 0.001)<br>**Men**: HR 1.69 (1.63–1.76; P < 0.001)<br>**Subtypes (HR, 95% CI):**<br>- Lip/oral cavity/pharynx: 2.10 (1.66–2.17)<br>- Respiratory organs: 1.91 (1.74–2.10)<br>- Female genital organs: 1.86 (1.56–2.17)	P < 0.001 in total; P < 0.001 in women; P < 0.001 in men. Cancer Subtype HRs (All P < 0.001):	Matched by age, gender, diabetes, obesity, and consultation frequency (no multivariate adjustment reported)

															- Skin: 1.83 (1.72–1.94)<br>- Lymphoid/ hematopoietic tissue: 1.77 (1.63–1.91)<br>- Digestive tract: 1.75 (1.64–1.87)<br>- Breast: 1.67 (1.52–1.84)<br>- Urinary tract: 1.64 (1.48–1.81)<br>- Male genital organs: 1.52 (1.40–1.66)		
Sagastagoitia-Fornieet et al. (2022)	Spain; Observational single-center cohort; Jan 2010 - Dec 2019 (median follow-up 4.07 years)	HF patients : 1,909	Mean= 64.4 (15.9 – 94.2)	Compared to general population	28.1%	Not Available (General population reference)	51.0 - 61.2%	53.3 - 56.1%	29.4 - 35.5 %	32.6 - 50.0 %	COPD: 9.8 – 15.2%;<br><br>Atrial fibrillation : 24.6 – 30.3%	By age/gender ; LVEF ≤40% vs >40%	165 new cancers (excluding skin); IR 1872/100,000 patient-years	Lung (23.6%), Colorectal (11.5%), Prostate (10.9%), Lymphoma (9.7%), Breast (6.1%), Bladder (5.5%)	Multivariable predictors wi thin HF cohort :<br>• Age: HR 1.04 (1.03–1.05) [<0.01]<br>• Smoking: HR 1.69 (1.19–2.38) [<0.01]<br>• ACE inhibitor: HR 1.56 (1.11–2.19) [0.01]	<0.01 (age, smoking); 0.01 (ACEi)	Age, smoking, COPD, CAD, gender, ACEi (competing-risk regression
Sakamoto et al. (2017)	Japan; Retrospective, single-center, cohort study (Jan 2001–Apr 2015)	Total: 5,238 HF patients	Mean= 64 ±12	Not reported	4.14%	Not Available (control: general population)	only "hypertensive heart disease" as baseline: 3.1%	Not reported	Not reported	Not reported	**Baseline diseases**:<br><br>Valvular heart disease: 2024 (38.6%)<br><br>Dilated cardiomyopathy: 642 (12.3%)<br><br>Hyper-	By BNP: ↑cancer prevalence with higher BNP (P = 0.042) By left ventricular ejection	.-CHF cohort: 198/5,238 **(3.78%)**<br>-General population (control): 749 767/127 692 000 (0.59%) (P < 0.0001).<br>**NOTE**: In the article, the prevalence was adjusted	•**Stomach cancer**: 0.90% (47/5238 in CHF vs. 0.10% (122,910/ 127 692 000) in control (9× higher prevalence).<br>•**Breast cancer**: 0.44% (23/5238) in CHF vs. 0.05% (65,085/127 6	Not provided (prevalence ratio: 3.85× higher in CHF) [Prevalence Ratio= Adjusted Prevalence in CHF/Prevalence in Control = 2.27%/0.59% = 3.85]	Not provided (prevalence ratio: 3.85× higher in CHF)	Age- and gender-adjusted prevalence (no multivariate HR)

											trophic cardio-myopathy: 362 (6.9%)	fraction (LVEF): No association (P = 0.338)<br>By LVDd: ↑cancer with smaller LVDd (P = 0.002)	to the Age/Sex): CHF cohort: 2.27% (95% CI: 1.89–2.71)<br>Control (general population): 0.59%	92 000) in control (8.8× higher).<br>•**Lung cancer**: 0.40% (21/5238,) in CHF vs. 0.08% (97,343/127 692 000) in control (5.0× higher).<br>•**Colon cancer**: 0.38% (20/5238) in CHF vs. 0.09% (112,772/127 692 000) in control (4.2× higher).<br>•**Prostate cancer**: 0.32% (17/5238) in CHF vs. 0.04% (51,534/127 692 000) in control (8× higher).<br>•**Other cancer** [cervical cancer, sigmoid colon cancer, renal cancer, uterine body cancer, liver cancer, esophageal cancer, rectal cancer, thyroid cancer, bile duct cancer and bladder cancer] 1.34% (70/5238) in CHF vs. 0.24% (300,123/127 692 000) in			

														control (5.6× higher).			
Yoshihisa et al. (2019)	Japan; Prospective observational study. Enrolled between 2010 and 2016	Total participants: 2,103 hospitalized HF patients.	Prior-CA group: 73.3 ±11.3<br><br>Non-prior-CA group: 66.1 ±14.8	N/A (all patients had HF)	Prior-CA group: 40.0%<br><br>Non-prior-CA group: 38.5%	N/A (no non-HF control group)	Prior-CA group: 74.9%<br><br>Non-prior-CA group: 71.1 %	Prior-CA group: 70.2%<br><br>Non-prior-CA group: 72.4%	Prior-CA group: 41.5 %<br><br>Non-prior-CA group: 40.5 %	Not reported (but CAD) prevalence was 32.7% vs. 33.2%	**Prior-CA vs. Non-prior-CA:**<br><br>CKD: 178 (64.7) vs. 982 (53.7) (P = 0.001)<br><br>Anemia: 182 (66.2)% vs. 929 (50.8) (P < 0.001)<br><br>Atrial fibrillation: 122 (44.4) vs. 690 (37.7) (P = 0.036)<br><br>COPD: 81 (29.5) vs. 404 (22.1) (P = 0.007)	**HF Subtypes**:<br>• HFrEF: Prior-CA associated with higher all-cause mortality (P < 0.001)<br>• HFpEF: Prior-CA associated with higher cardiac events (P = 0.011)	New cancer post-HF: 6.2% in both groups (17/275 (6.2%) prior-CA vs. 114/1,828 (6.2%) non-prior-CA)	**Most frequent cancers:**<br>• Prior-CA: Stomach, colorectum, blood/lymph, prostate, breast, lung<br>• New post-HF: Stomach, colorectum, lung, blood/lymph, liver<br>• CA-related deaths: Liver, stomach, lung, blood/lymph, colorectum	Prior CA was a predictor of cardiac event rate (HR 1.450, 95% CI 1.134–1.822) , P=0.001,and all-cause death (HR 2.483, 95% CI 2.034–3.030), P < 0.001	**Cardiac Events:**<br><br>HR: 1.450 (95% CI: 1.154–1.822)<br><br>p-value: P = 0.001 (from univariate Cox analysis)<br><br>**All-Cause Death:**<br><br>HR: 2.483 (95% CI: 2.034–3.030)<br><br>p-value: P < 0.001	Not fully adjusted (subgroup analysis only; no multivariate Cox model due to collinearity)

HF = heart failure; DM = diabetes mellitus; MI = myocardial infarction; SNDS = Système National des Données de Santé; COPD = chronic obstructive pulmonary disease; CHF = congestive heart failure; CA = cancer; CAD = coronary artery disease; LVEF = left ventricular ejection fraction; LVDd = left ventricular diastolic diameter; BNP = B-type natriuretic peptide; IR = incidence rate (per 100,000 person-years); HR = hazard ratio; sHR = sub-distribution hazard ratio; CI = confidence interval.

"Not reported" indicates that the original study did not provide the corresponding data. "Not available" signifies that a non-HF control group was not included (e.g., comparison with general population estimates or no comparator).
