# Supplementary material for: The Association Between Cancer Incidence and Heart Failure: A Systematic Review and Meta-Analysis
Source: Diagnostics (Basel). 2026 Jun 28;16(13):2016. doi: 10.3390/diagnostics16132016 (PMC13359713; doi:10.3390/diagnostics16132016)
Supplement: Supplementary file 1 [file diagnostics-16-02016-s001.zip › Supplementary File S3 (Quality Assessment of the Included Studies).pdf]

Quality Assessment of the Included Studies

**Study**	**S1**	**S2**	**S3**	**S4**	**C1**	**C2**	**O1**	**O2**	**O3**	**Total /9**	**Remarks**
Banke et al., 2016[12]	√	√	√	√	√	X	√	√	√	**8/9**	C2: No adjustment for smoking, alcohol, BMI
Mirabel et al., 2025[3]	√	√	√	√	√	√	√	√	√	**9/9**	Comprehensive adjustment including tobacco, alcohol, obesity
Bruhn et al., 2023[2]	√	√	√	√	√	X	√	√	√	**8/9**	C2: No adjustment for BMI, smoking, alcohol
Roderburg et al., 2021[4]	√	√	√	X	√	X	√	√	√	**7/9**	S4: No early-cancer exclusion period; C2: No multivariate adjustment for smoking/alcohol
Sagastagoitia-Fornie et al., 2022[14]	√	X	√	√	X	X	√	√	√	**6/9**	S2: No non-HF control group (compared to general population estimates); C1/C2: No comparison group
Sakamoto et al., 2017[13]	√	X	√	X	√	X	√	√	X	**6/9**	S2: No non-HF control; S4: No exclusion of prevalent cancer; C2: No adjustment for smoking, alcohol, BMI, diabetes, medications, or socioeconomic status; O1: Outcome is cumulative incidence proportion, not incidence rate; O3: No individual follow-up duration reported
Yoshihisa et al., 2019[15]	√	X	√	X	X	X	√	√	√	**5/9**	S2: No non-HF control group; S4: No early-cancer exclusion period; C2: Confounders analyzed one by one, not adjusted together

S1=Representativeness of HF cohort, S2= Selection of the non-exposed cohort, S3= Ascertainment of HF diagnosis, S4= Demonstration that cancer was not present at baseline, C1= Control for age/sex, C2= Control for additional confounders, O1=Cancer assessment method, O2=Follow-up Length O3=Follow-up Adequacy.

NOTES

Some cancers not typically related to HF—such as non-melanoma skin cancer, thyroid cancer, and localized prostate cancer—were not excluded, which may overestimate the overall cancer risk

NMSC Handling

**Study**	**NMSC Handling**	**Notes**
Banke et al., 2016[12]	Included (not excluded)	All types of cancer included" (ICD-10 C00–C96) — NMSC (C44) falls within this range
Mirabel et al., 2025[3]	Included (but lower risk observed)	Skin cancers were captured; lower risk in HF patients; no explicit exclusion stated
Bruhn et al., 2023[2]	Analysed separately	Verified
Roderburg et al., 2021[4]	Included in overall cancer incidence	Verified
Sagastagoitia-Fornie et al., 2022[14]	Excluded from analysis	Explicitly states "excluding non-melanoma skin cancer"
Sakamoto et al., 2017[13]	Not reported	Reports specific cancers (stomach, lung, prostate, breast, colon); NMSC not mentioned
Yoshihisa et al., 2019[15]	Not reported	NMSC not discussed

Note: For Banke (2016) and Mirabel (2025), NMSC was not explicitly excluded; skin cancers were included in the overall cancer definition. However, Mirabel et al. reported a lower risk of skin cancers in HF patients, suggesting minimal inflationary bias.
