# Supplementary material for: The Association Between Cancer Incidence and Heart Failure: A Systematic Review and Meta-Analysis
Source: Diagnostics (Basel). 2026 Jun 28;16(13):2016. doi: 10.3390/diagnostics16132016 (PMC13359713; doi:10.3390/diagnostics16132016)
Supplement: Supplementary file 1 [file diagnostics-16-02016-s001.zip › Supplementary File S4 (Sensitivity Analysis).pdf]

Sensitivity analysis (Leave one out study)

1)

Study or Subgroup	log[Hazard Ratio]	SE	Weight	Hazard Ratio IV, Random, 95% CI
Banke 2016	0.215	0.037	0.0%	1.24 [1.15, 1.33]
Mirabel 2025	0.058	0.008	50.1%	1.06 [1.04, 1.08]
Roderbur 2021	0.565	0.026	49.9%	1.76 [1.67, 1.85]
**Total (95% CI)**			**100.0%**	**1.36 [0.83, 2.24]**

Heterogeneity: $Tau^2 = 0.13$; $Chi^2 = 347.36$, df = 1 ($P < 0.00001$); $I^2 = 100\%$
Test for overall effect: Z = 1.23 (P = 0.22)

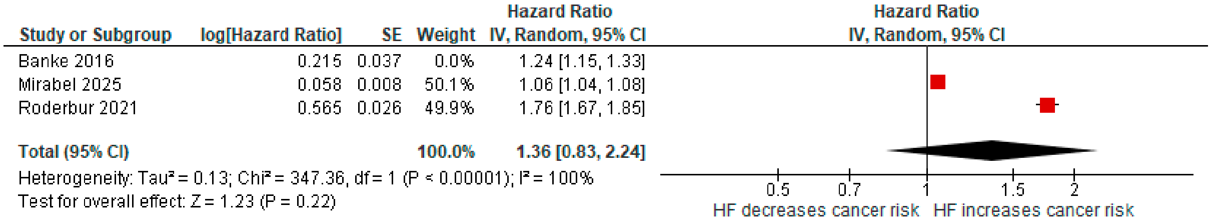

2)

Study or Subgroup	log[Hazard Ratio]	SE	Weight	Hazard Ratio IV, Random, 95% CI
Banke 2016	0.215	0.037	49.7%	1.24 [1.15, 1.33]
Mirabel 2025	0.058	0.008	0.0%	1.06 [1.04, 1.08]
Roderbur 2021	0.565	0.026	50.3%	1.76 [1.67, 1.85]
**Total (95% CI)**			**100.0%**	**1.48 [1.05, 2.08]**

Heterogeneity: $Tau^2 = 0.06$; $Chi^2 = 59.90$, df = 1 ($P < 0.00001$); $I^2 = 98\%$
Test for overall effect: Z = 2.23 (P = 0.03)

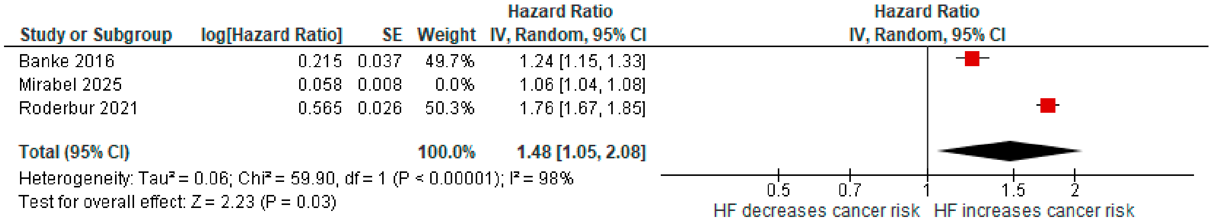

3)

Study or Subgroup	log[Hazard Ratio]	SE	Weight	Hazard Ratio IV, Random, 95% CI
Banke 2016	0.215	0.037	47.4%	1.24 [1.15, 1.33]
Mirabel 2025	0.058	0.008	52.6%	1.06 [1.04, 1.08]
Roderbur 2021	0.565	0.026	0.0%	1.76 [1.67, 1.85]
**Total (95% CI)**			**100.0%**	**1.14 [0.98, 1.33]**

Heterogeneity: $Tau^2 = 0.01$; $Chi^2 = 17.20$, df = 1 ($P < 0.0001$); $I^2 = 94\%$
Test for overall effect: Z = 1.69 (P = 0.09)

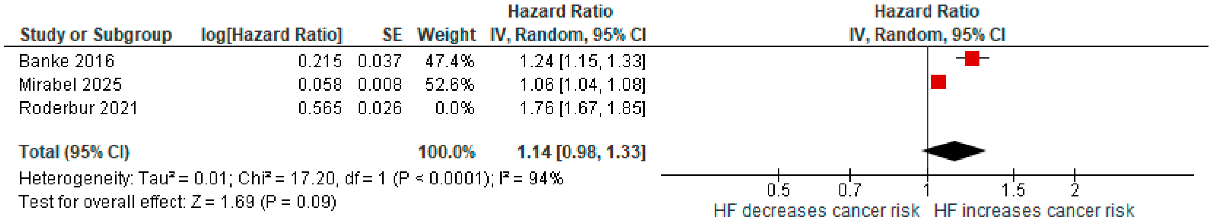

Supplementary File S4. Leave-one-out sensitivity analysis for the association between heart failure and incident cancer.

Random-effects meta-analysis of three cohort studies, iteratively omitting one study at a time. Exclusion of Mirabel (2025) produced a significant pooled HR (1.48, 95% CI 1.05–2.08; p = 0.03). Exclusion of Roderburg (2021) resulted in a non-significant HR (1.14, 0.98–1.33; p = 0.09). Exclusion of Banke (2016) also yielded a non-significant HR (1.36, 0.83–2.24; p = 0.22). Heterogeneity remained high in all iterations (I^2 = 94–100%). SE=standard Error, IV= inverse variance, and HF= heart failure
